# A NEW NORMAL: AARP SURVEY FINDINGS ON OLDER ADULTS’ EXPERIENCES AND PERSPECTIVES ON TELEHEALTH AND AI IN HEALTH CARE

**DOI:** 10.1093/geroni/igae098.0086

**Published:** 2024-12-31

**Authors:** Cheryl Lampkin, Teresa Keenan

**Affiliations:** AARP, Washington, District of Columbia, United States; AARP, Washington, District of Columbia, United States

## Abstract

The COVID-19 pandemic ushered in a “new normal” as it relates to receiving health care--especially for older adults. This presentation will include insights on how new technology and attitudes about technology have impacted how older adults receive health care services. For example, findings from a recent survey of 1,700 older adults conclude that while over half say they are likely to attend a phone or video session with a mental health professional (53%) or their primary care provider (52%) for a mental health concern, most are skeptical of telehealth believing they get better quality care for both physical (82%) and mental health (69%) concerns in person. Similarly, another AARP study found older adults believe in-person visits are better than telehealth visits when it comes to providing a personal touch (73%), diagnosis accuracy (72%), and thoroughness (68%). However, many said in-person and telehealth are about the same when it comes to keeping personal information safe (60%) and private (52%). These and other insights are based on data obtained from multiple recent AARP surveys fielded among nationally representative samples of midlife and older adults. Studies also included oversamples of Black/African American and Hispanic/Latino respondents, enabling enhanced analyses. This multi-analysis presentation will provide unprecedented insights on familiarity and use of technology in receiving health care as well as opinions and attitudes on the use of artificial intelligence (AI) in health care. Additionally, a preview of new data collected in late fall 2024 will provide timely insights on current views on health care innovations.

